# Leprosy New Case Detection Trends and the Future Effect of Preventive Interventions in Pará State, Brazil: A Modelling Study

**DOI:** 10.1371/journal.pntd.0004507

**Published:** 2016-03-03

**Authors:** Haroldo José de Matos, David J. Blok, Sake J. de Vlas, Jan Hendrik Richardus

**Affiliations:** 1 Instituto Evandro Chagas, Belém, Pará, Brazil; 2 Department of Public Health, Erasmus MC, University Medical Center Rotterdam, Rotterdam, The Netherlands; Baylor College of Medicine, UNITED STATES

## Abstract

**Background:**

Leprosy remains a public health problem in Brazil. Although the overall number of new cases is declining, there are still areas with a high disease burden, such as Pará State in the north of the country. We aim to predict future trends in new case detection rate (NCDR) and explore the potential impact of contact tracing and chemoprophylaxis on NCDR in Pará State.

**Methods:**

We used SIMCOLEP, an existing individual-based model for the transmission and control of *M*. *leprae*, in a population structured by households. The model was quantified to simulate the population and observed NCDR of leprosy in Pará State for the period 1990 to 2014. The baseline scenario was the current control program, consisting of multidrug therapy, passive case detection, and active case detection from 2003 onwards. Future projections of the NCDR were made until 2050 given the continuation of the current control program (*i*.*e*. baseline). We further investigated the potential impact of two scenarios for future control of leprosy: 1) discontinuation of contact tracing; and 2) continuation of current control in combination with chemoprophylaxis. Both scenarios started in 2015 and were projected until 2050.

**Results:**

The modelled NCDR in Pará State after 2014 shows a continuous downward trend, reaching the official elimination target of 10 cases per 100,000 population by 2030. The cessation of systematic contact tracing would not result in a higher NCDR in the long run. Systematic contact tracing in combination with chemoprophylaxis for contacts would reduce the NCDR by 40% and bring attainment of the elimination target two years forward to 2028.

**Conclusion:**

The NCDR of leprosy continues to decrease in Pará State. Elimination of leprosy as a public health problem could possibly be achieved around 2030, if the current control program is maintained. Providing chemoprophylaxis would decrease the NCDR further and would bring elimination forward by two years.

## Author Summary

Leprosy remains a public health problem in Brazil. With over 30,000 new cases detected annually, it has the second-largest number of leprosy cases detected worldwide. Although the overall number of new cases is declining, there are still areas with a high disease burden, such as Pará State. In this study, we used the individual-based model SIMCOLEP to predict the future trend of the new case detection rate of leprosy (NCDR) in Pará State given the current control strategy. Additionally, we explored the potential impact of contact tracing and chemoprophylaxis on the NCDR. Our results show that the NCDR continues to decrease in Para State, reaching elimination around 2030. Contact tracing alone would not further reduce the NCDR. However, when contact tracing is combined with chemoprophylaxis the NCDR would be reduced up to 40% and elimination could be brought forward by two years.

## Introduction

Leprosy, also known as Hansen Disease, is caused by *Mycobacterium leprae*. Every year more than 210,000 new cases are detected worldwide [1]. After India, Brazil has the second largest number of leprosy patients detected. In 2014, there were 31,064 new cases reported, mostly from the Amazon region [1]. Brazil was unsuccessful in bringing the prevalence of leprosy below the official WHO’s ‘elimination as public health problem’ goal of less than 10 cases per 100,000 population by the year 2000 [2]. In 2010 the leprosy prevalence rate in Brazil was 15.6 per 100,000 and the new case detection rate (NCDR) 18.2 per 100,000, although the NCDR had dropped by about 35% between 2001 and 2010 [3].

In 2011, the Ministry of Health of Brazil (MHB) had set the year 2015 as the new target year to achieve elimination of leprosy [3]. This target is now part of the agenda of an ambitious governmental program for reducing poverty in Brazil, which is called “Brasil sem miséria” (*Brazil without misery)* [3]. The spatial distribution of leprosy in Brazil is known to be heterogeneous [4, 5]. Therefore, the MHB selected 243 municipalities as priority for actions aimed at reducing the leprosy burden in order to achieve the elimination target by 2015. Most of these municipalities are located in the Amazon region, and Pará State alone has 20% of all priority municipalities [3].

The NCDR of leprosy in Brazil between 1990 and 2010 shows a characteristic trend of an increasing rate up to 2003 followed by a gradual decrease afterwards. A similar trend can also be observed in Pará State, but with a NCDR that is more than two times higher (45.8 per 100,000 in 2014) [6]. Based on a simple extrapolation of the NCDR trend, policy makers expect elimination of leprosy to be achieved by 2015 in Brazil as a whole, but this will certainly not be the case for Pará State. Furthermore, simple extrapolation of trends fails to take into account dynamic processes that underlie the observed trend, such as the importance of contacts and in particular of households for the transmission of *M*. *leprae* [7].

To assess the future leprosy NCDR trend in Pará State in the Amazon Region of Brazil, we will apply the individual-based (or microsimulation) model SIMCOLEP [8]. It models the transmission and control of leprosy in a population structured by households and can be used to explore the potential impact of interventions targeted at households [8]. SIMCOLEP has been used to study the leprosy epidemic in northwest Bangladesh, India, Brazil and Indonesia [8, 9]. It has also been applied to assess the various strategies for leprosy control such as contacts tracing, the effect of Bacillus Calmette-Guérin (BCG) vaccination in infants, and also possible future strategies such as chemoprophylaxis and early diagnosis of subclinical infection [10]. The impact of such targeted strategies is estimated in terms of leprosy incidence at the population level [8, 10]. We therefore also address the question to which extent contact tracing and chemoprophylaxis for leprosy contacts contribute to the elimination of leprosy in Pará State. In summary, the aims of this study are: 1) to predict future trends in the NCDR; and 2) to explore the potential impact of contact tracing and chemoprophylaxis on the NCDR in Pará State.

## Methods

SIMCOLEP models the transmission and control of *M*. *leprae* in a population structured by households [8]. It simulates life histories of individuals and the natural history of leprosy. Life histories are described by birth, death, and movement between households. Individuals are placed in households that are formed, change and dissolve during the simulation. Individuals can move from one household to another or create an own household after marriage or during adolescence. Children, which are placed in the household of their mother, are only born to married couples [8, 11]. The population grows following the population growth rate of Pará State. Birth and deaths are determined using fertility rates and death rates. Demographic data were obtained from the Brazilian Institute for Geography and Statistics (IBGE) [12, 13].

In the model, only susceptible individuals will progress to leprosy. We assume that 80% of the population is not susceptible to leprosy [8, 14]. Since the underlying mechanism of susceptibility of leprosy is still unknown, we assume that each individual has an equal probability of being susceptible (*Random mechanism*) [8]. Transmission of *M*. *leprae* occurs when an infectious individual has contact with a susceptible individual. Contacts are made with individuals 1) in the general population at rate *c*_*pop*_ and 2) within households at rate *c*_*hh*_. Infectivity is determined by multiplying the contact rate with the probability of infection during a contact. The model distinguishes two types of leprosy: self-healing, which is considered equivalent to paucibacillary leprosy (PB), or chronic, which is considered equivalent to multibacillary leprosy (MB). Both types can be detected, treated and cured, but only chronic infections are considered infectious in the model. The natural history of leprosy is modelled following the model of Meima *et al*. [14]. Based on surveillance data from ministry of Health, for Pará State, we assumed that around 60% of the susceptible population undergoes a chronic infection (MB) and 40% a self-healing infection (PB) when infected [3].

The leprosy control program in Pará State includes treatment, both passive and active case detection activities, and BCG vaccination, which is recommended by the Brazilian Ministry of Health for household contacts [3]. In the model, treatment was started in 1970, which included Dapsone monotherapy. This was replaced by multidrug therapy (MDT) after 1989 [15]. Passive case detection is reflected by detection delays. The average detection delay was estimated to decrease from 18 years in 1970 to 3 years in 2000s. Active contact tracing started in 2003, with the Family Health Program, but has not been completely implemented to date [3]. The coverage of contact tracing was set to 43% in 2003 to 59% after 2010, meaning that after examination 59% of symptomatic cases will be detected [16]. BCG vaccination is assumed to have a protective effect of 60% against leprosy [17, 18]. The BCG vaccination program for infants to prevent severe TB cases started in 1975. The coverage is around 99% since 2000 [19].

### Calibration

The modelled household structures in the population were fitted to the observed distribution of household size in Brazil, obtained from IBGE [12, 13]. In order to fit the household structure, parameters involving movements of individuals between households were calibrated (see Table 1). We only calibrated the following parameters: fraction of non-married males moving, fraction of individuals moving to child after becoming widowed, fraction of moving individuals that create their own household, and the weighting function to determine the household size to which individuals move. For all other parameters, we assumed that they would be similar to previous modelling work [8]. The simulated distribution of household size did not show any significant difference with the observed distribution (p>0.05; χ^2^-test).

**Table 1:** Overview of calibrated parameters.

*Parameters*	*Values*
**Household movements**	
- Fraction of non-married individuals moving randomly	Males: 0.75^a^
	Females: 0.0 ^b^
- Age of random movements of non-married individuals	12–22 years ^b^
- Household size to move to	Triangular distribution (0,4,2)^a^
- Rate at which households are split after adding a married couple	Exponential(12) ^b^
- Fraction of individuals moving to child after becoming a widow/widower	Males: 1.0^a^
	Females: 1.0^a^
- Fraction moving to partner after marriage	Males: 0.0 ^b^
	Females: 0.75 ^b^
- Fraction of moving person that create their own household	Males: 0.01^a^
	Females: 0.0 ^b^
**Leprosy**	
- Detection delay	1970–1980: 18 years ^b^
	1981–1990: from 18 to 16 years ^b^
	1991–2000: from 16 to 10.5 years ^b^
	2001–2010: from 10.5 to 4.5 years ^b^
	After 2011: 3 years ^b^
- Contact rate in the general population	0.543 (95% CI: 0.503–0.584) ^a^
- Contact rate within households	0.98 ^b^

^a^ Calibrated (See Blok et al. 2015 [11]);

^b^ Optimal values obtained from Fischer et al. (2010) [10];

Subsequently, we fitted the leprosy situation of the model to the observed NCDR in Pará State between 1990 and 2014. NCDR data were obtained from the *Sistema de Informações de Agravos de Notificação* (Sinan) database [6, 20]. Sinan is a national database for reporting communicable diseases in Brazil. We only calibrated detection delays and the contact rate in the general population (*c*_*pop*_) [9]. Detection delays were estimated following the NCDR trend in the data. As a starting point the most recent detection delay was set to 3 years [21]. Table 1 provides an overview of the estimated delays over time. The contact rate in the general population was calibrated such that the modelled NCDR would match the observed NCDR between 1990 and 2014. The contact rate within households (*c*_*hh*_) was set to the optimal value (*c*_*hh*_ = 0.98) of previous work [8, 9]. The best fit was determined by a log-likelihood function assuming a Poisson distribution. The model outcomes are based on an average of 100 simulation runs. A detailed description of the fitting procedure is found in Fischer *et al*. [8].

### Predictions & intervention scenarios

Simulations were run until the year 2050 to predict the future trends in leprosy. The baseline scenario represents the current leprosy situation and the existing control program as described above in Pará State. Additionally, we investigated the potentially impact of two scenarios of future control of leprosy: 1) discontinuation of contact tracing; 2) continuation of current control (including contact tracing) in combination with chemoprophylaxis. The first scenario was tested, because in practice coverage rates of contact tracing easily fluctuates [16]. To assess the importance of contact tracing alone for transmission, we tested to which extent discontinuation of contact tracing (an extreme measure) would potentially impact the NCDR trend in Pará State. In the second scenario, we assessed the impact of administering chemoprophylaxis to contacts given the continuation of contact tracing. Chemoprophylaxis (i.e. single dose of Rifampicin) was only given once after examination, assuming that it cures 50% of subclinical cases [22]. We further assumed that the probability for an individual to comply was 75%. Both scenarios started in 2015 and were projected until 2050.

## Results

**Fig 1:**
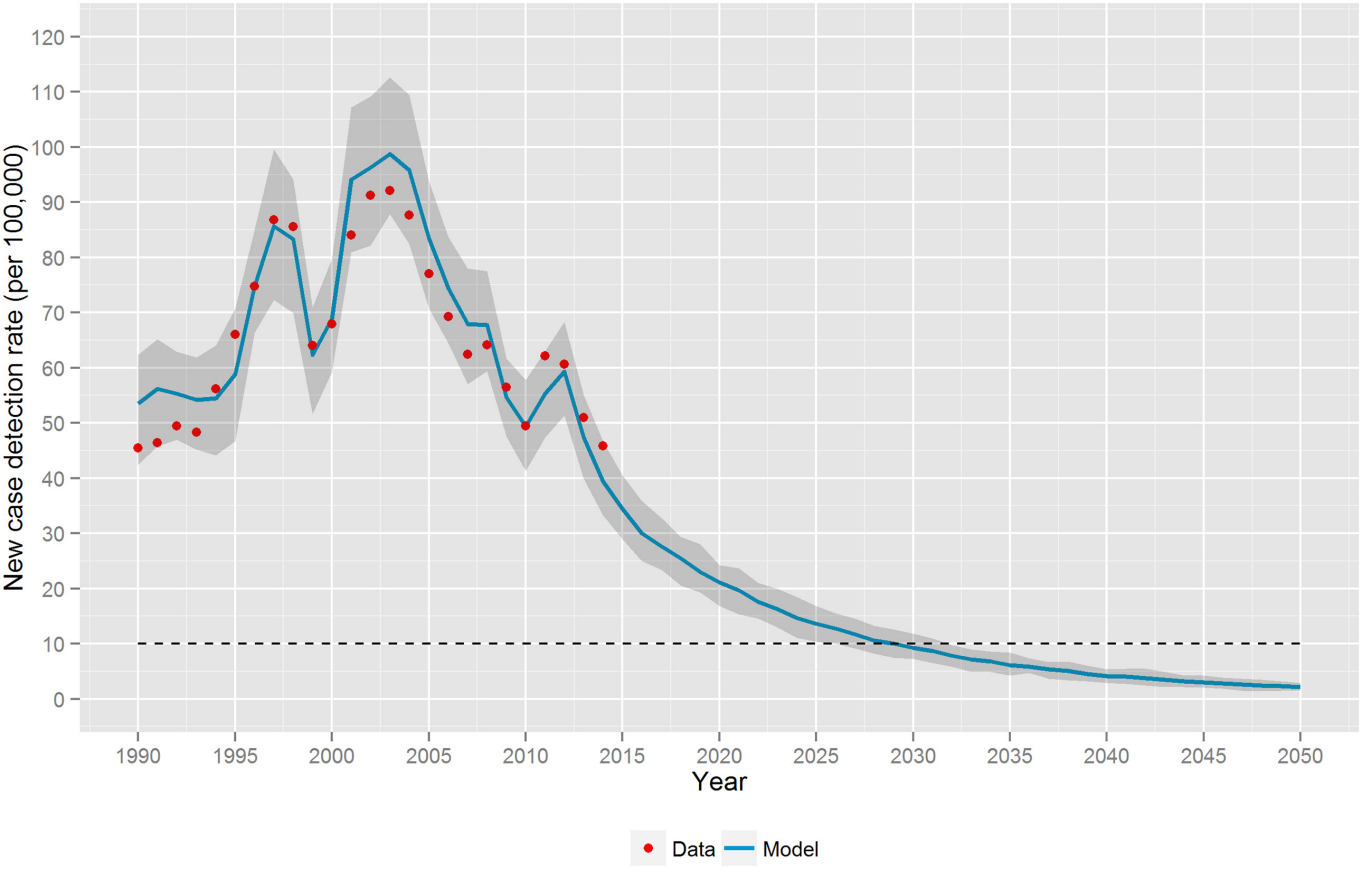
Projections of the new case detection rate in in Pará State, Brazil from 1990 to 2050. The model was able to fit the observed data (1990–2014). Results are the average of 100 runs. The shaded area represents the confidence interval (stochastic variation between individual runs).

Fig 1 shows the results of the simulated leprosy NCDR against the observed NCDR in Pará State between 1990 and 2014. The fitted values are found in Table 1. The simulation provided a good fit to the observed data, and based on this fit it was possible to predict the future trend up to 2050. Our prediction shows that the NCDR in Pará State continues to decline with an annual decrease of 8% to 2.17 per 100,000 cases (90% CI: 1.48–2.89) in 2050. This corresponds with a drop in terms of annual new cases to around 400 new cases. The elimination target (10 cases per 100,000 population) would be reached around 2030.

**Fig 2:**
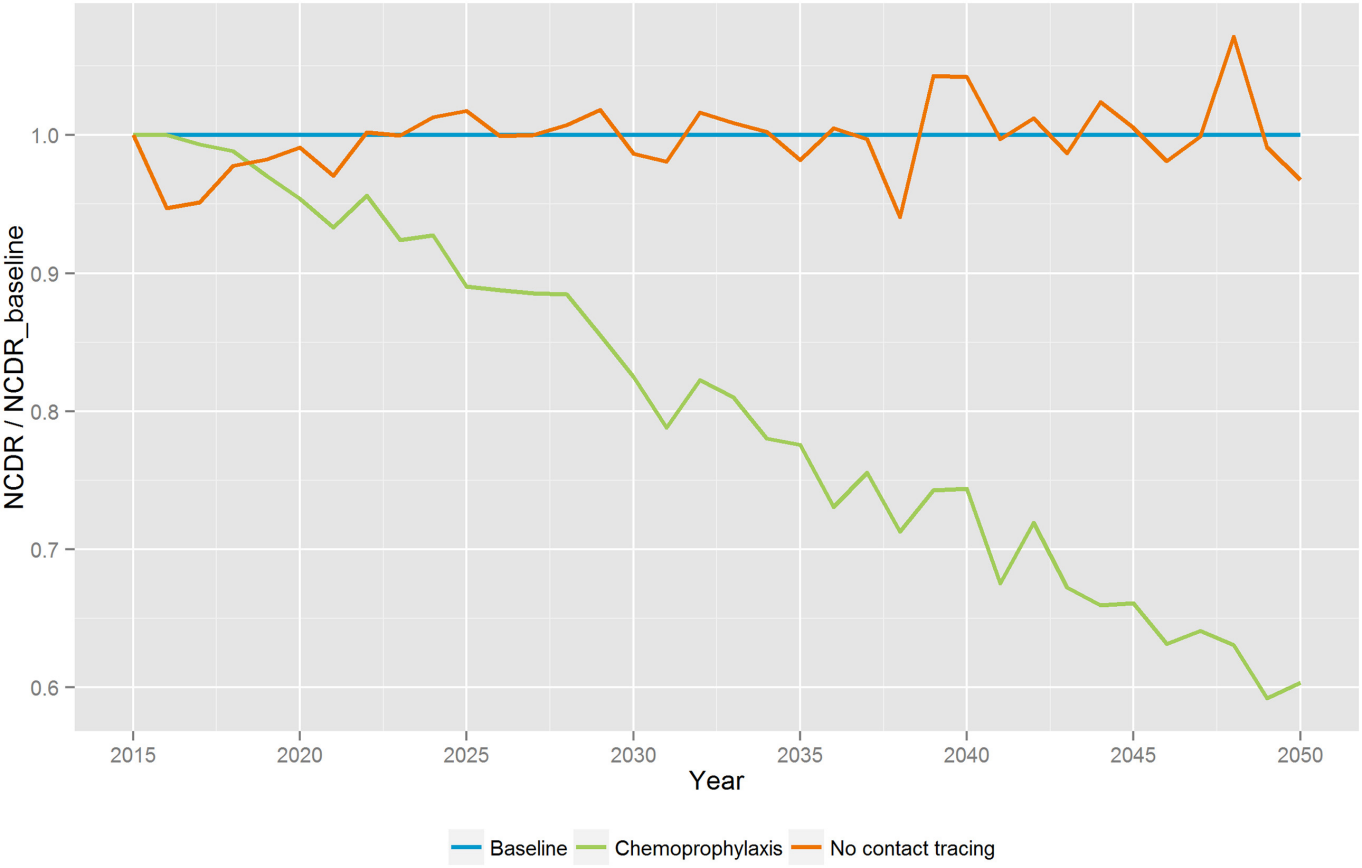
Predicted impact of two scenarios of future leprosy control: 1) discontinuation of contact tracing, and 2) contact tracing in combination with chemoprophylaxis. The figure presents the relative difference in new case detection rates in comparison with the prediction from the baseline scenario. Results are an average of 100 runs.

Discontinuation of contact tracing in Pará State would not increase the NCDR in the long run relative to the baseline scenario (Fig 2). In the first years a drop in the NCDR is observed, because more cases are missed as result of the discontinuation of contact tracing. These cases are expected to be detected at a later stage through passive detection. Afterwards, it slowly rises to the level of the baseline scenario. The relative impact of chemoprophylaxis compared to the baseline scenario shows a substantial impact on the NCDR of leprosy. In the long run the predicted NCDR is up to 40% lower than the baseline. The NCDR drops immediately below the level of the baseline scenario due to the cured subclinical cases. Over time the effect increases because it would prevent new infections.

**Fig 3:**
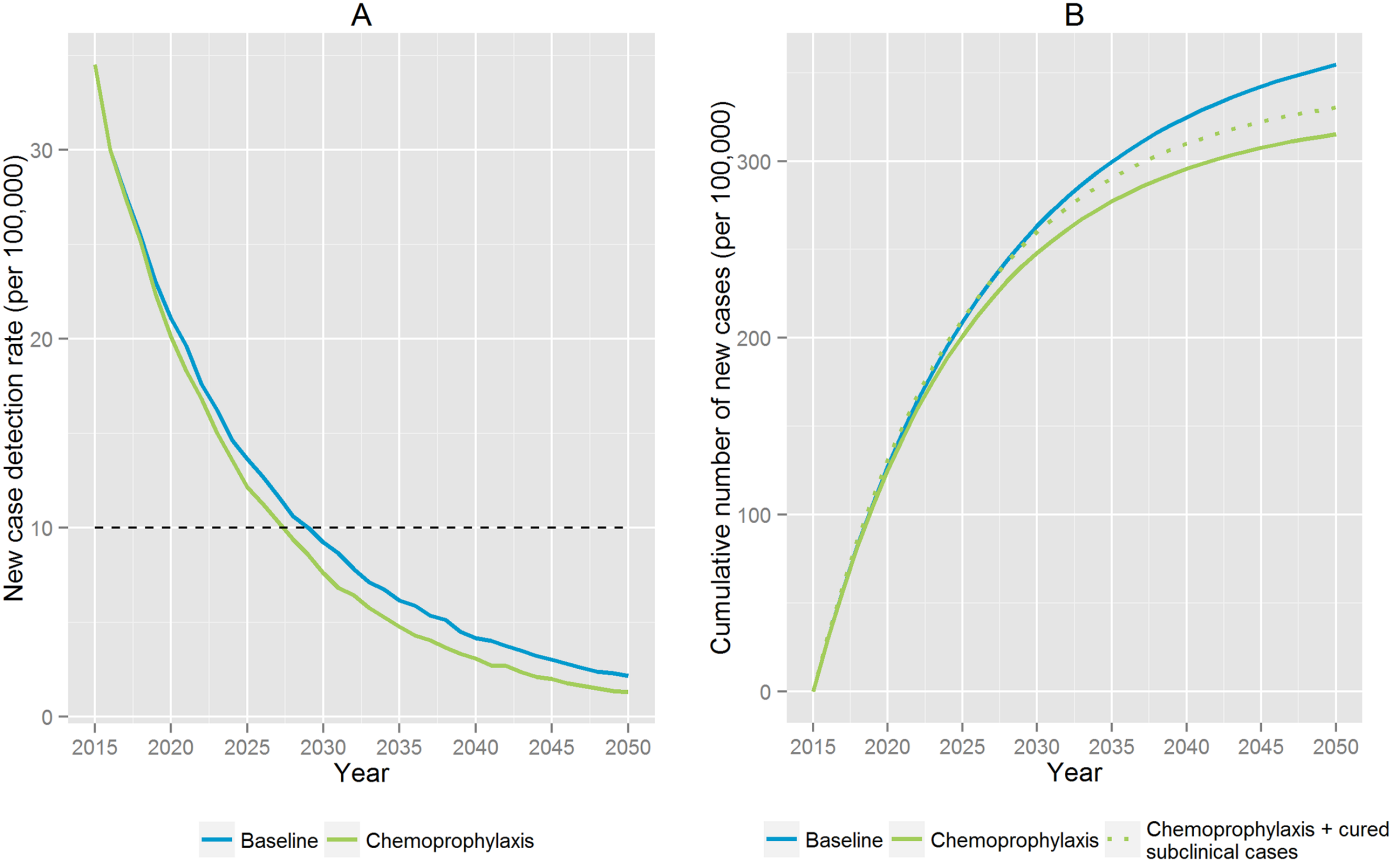
Impact of chemoprophylaxis on the new case detection rates of leprosy in Pará State, Brazil. A) Predicted decline of the new case detection rates with chemoprophylaxis since start of the intervention. B) Cumulative new cases detected (per 100,000 person population) since start of the intervention. Results are an average of 100 runs.

Fig 3A and 3B focus on the impact of chemoprophylaxis in comparison with the baseline. The NCDR of leprosy is predicted to decrease to around 1.13 per 100,000 (90% CI: 0.50–1.98) in 2050, indicating an annual decrease of 9.5% (Fig 3A). Moreover, the elimination target would be reached around 2028, which is two years earlier than in the baseline scenario. Fig 3B shows the cumulative number of new cases detected. In total 40 new cases per 100,000 could be avoided over the whole period compared to the baseline. It also illustrates that chemoprophylaxis would prevent new infections on top of the cured subclinical infections over time.

## Discussion

The new case detection rate of leprosy in the whole country of Brazil as well as in Pará State has been showing a downward trend since 2005. With the SIMCOLEP model we were able to reproduce the observed NCDRs between 1990 and 2014. The modelled trend in Pará State after 2014 shows a continuous downward trend, reaching elimination (less than 1 per 10,000) by 2030. Systematic contact tracing alone would seem to have no additional impact on the NCDR in the long term, but contact tracing together with chemoprophylaxis for contacts would add a significant impact to the reduction of new cases in the long term.

Our model predictions underscore the importance of the maintenance of early diagnosis (i.e. short detection delay), treatment of newly found cases with MDT, and BCG vaccination coverage; these are our baseline conditions. We have shown that ceasing contact tracing will not change the NCDR trend much. Previous work found similar findings [10]. The reason for this is that patients are only found when they have symptoms, meaning that transmission might already have been taken place during the asymptomatic state. Hence, systematic contact tracing alone does not have a noticeable effect on the ongoing transmission of *M*. *leprae* and subsequent leprosy disease. However, contact tracing in itself will still benefit the individual with leprosy, because he or she will be detected and treated earlier. This would interrupt the disease process in most cases, preventing severe nerve damage (grade-2 disability) [23].

Systematic contact tracing, however, is effective when combined with an additional preventive intervention. Providing contacts with chemoprophylaxis showed to further reduce the number of new leprosy cases up to 40% in the long run. It also supports attaining the target of zero transmission in the population [24]. Chemoprophylaxis is yet to be introduced systematically in Brazil, but the results of our study indicate that this intervention will provide substantial benefit to the leprosy control program through reduced costs and disease burden. However, the feasibility to carry out this program on a large scale, such as in Pará State, remains a concern. The Ministry of Health is currently stimulating projects for evaluating contact chemoprophylaxis, but this is still on a small scale [2].

Our model predictions are based on the reported NCDRs in Brazil. The validity of our results therefore depends on the reliability of these reported data. In fact, the NCDR did not change much between 1990 and 2010; 2.0 per 10,000 in 1990 and 1.8 per 10,000 in 2010. During this period however, there was considerable variability in the trend, with marked increases and decreases. In Pará State, the NCDR was twofold higher than in Brazil as a whole: 4.6 per 10,000 in 1990 and 5.0 per 10,000 in 2010, and also showed marked variability [2, 3]. The variability in the trend is explained largely by operational factors influencing the leprosy control program, and these have been taken into account in the model. The gradual implementation of SINAN can fully explain the sharp increase in detected cases from 1990 to 1997, even though the underlying rate of all new cases (detected and undetected) may have remained the same. In the mid-1990s, another important intervention occurred in Brazil. Since 1994 the Family Health Program was introduced, with a focus on primary health care and health promotion, including active case finding as a main health surveillance methodology [25]. Leprosy, until then a disease diagnosed mainly by dermatologists, became an object for primary health care from 2000 onwards. This development is likely to be reflected in the second increase of the NCDR in the first decade of the current century, and particularly in Pará State. The sharp increase of the NCDR in the late 1990’s up to 2005 due to increased active case detection and registration indicates an enormous backlog of previously undetected cases that were now being found and treated. The reduction seen after 2014 would then be a return to a basic situation with equilibrium in terms of incidence and prevalence, but in a downward trend [25]. Our modelling results support the observed reduction in new case detection after 2014.

A remaining threat to the elimination of leprosy is missing cases. Previous work has shown that the prevalence of undiagnosed cases (missing cases) is substantial [26–28]. The data used in this study very likely underestimate the number of cases in Pará State. Because our predictions depend on available data, the problem of missing cases is also present in our predictions. It is likely that the actual number of new cases is higher than predicted.

Also, the coverage of contact tracing, which was set to 59% based on a report of the Ministry of Health [16], might be too optimistic. We therefore conducted a sensitivity analysis with a more realistic coverage of 40% to assess the impact of chemoprophylaxis on NCDR. Results showed that lowering the coverage would not affect our model predictions significantly (S1 Fig).

The results of our study not only depend on the validity of the data, but also on the availability of the data and the model´s underlying assumptions regarding leprosy. For example, data about the prevalence or incidence by household size and by contact were not available for Pará State. These data are needed to fit the contact rates within households directly. As a solution we used the optimal value from previous work [8], assuming that the rate at which people have effective contact with other household members would not differ much between countries and regions. Also, our model assumes that susceptibility was fully randomly determined. Previous work has shown that this is a valid assumption, but could not rule out other mechanisms, such as genetic inheritance [8]. It has also shown that assuming random susceptibility will provide the most optimistic results.

Our future projections did not account for any adverse events in the future, such as a famine, or any changes in leprosy control, which may alter the course of the epidemic. It is likely that in areas with declining NCDR, the incentive to find new cases may disappear [29].

Based on the observed data for Pará State, our modelling study confirms that the leprosy incidence appears to be decreasing in Brazil. Nevertheless, additional interventions should be implemented in view of the constant number of new cases detected in Pará state. Achievement of elimination could be brought forward with a number of years through systematic contact tracing combined with the application of chemoprophylaxis.

## Supporting Information

S1 Fig Impact of chemoprophylaxis on the new case detection rates of leprosy assuming a lower coverage of contact tracing (40%). (TIFF)
