# Supplementary figures and images for: Leprosy New Case Detection Trends and the Future Effect of Preventive Interventions in Pará State, Brazil: A Modelling Study
Source: PLoS Negl Trop Dis. 2016 Mar 3;10(3):e0004507. doi: 10.1371/journal.pntd.0004507 (PMC4777416; doi:10.1371/journal.pntd.0004507)

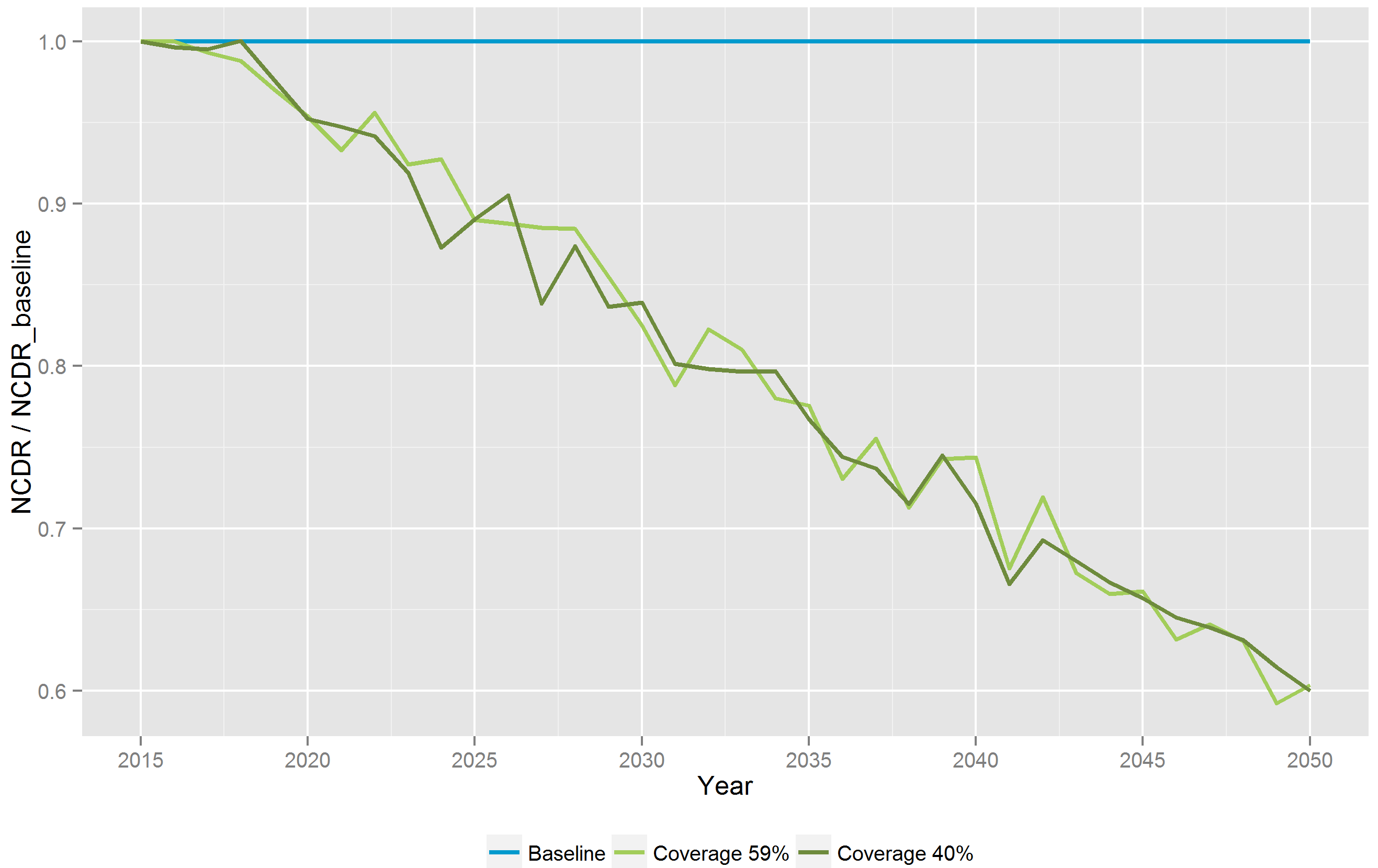

Supplement: S1 Fig — (TIFF) [file pntd.0004507.s001.tiff]
